# Targeting PI3K/AKT/mTOR Signaling Pathway in Breast Cancer

**DOI:** 10.3390/cancers13143517

**Published:** 2021-07-14

**Authors:** Huayi Li, Lorenzo Prever, Emilio Hirsch, Federico Gulluni

**Affiliations:** Department of Molecular Biotechnology and Health Sciences, University of Turin, 10126 Turin, Italy; huayi.li@unito.it (H.L.); lorenzo.prever@unito.it (L.P.); emilio.hirsch@unito.it (E.H.)

**Keywords:** PI3K, inhibitor, AKT, mTOR, breast cancer, clinical trial, metastasis, class II PI3K

## Abstract

**Simple Summary:**

PI3K signaling pathway plays an essential role in many cellular processes and is frequently altered in breast cancer, leading to increased tumor growth and reduced survival. Small molecule inhibitors have been developed that target the three key elements of this pathway: PI3K, AKT, and mTOR. Despite demonstrating promising preclinical activity, intrinsic and acquired resistance, as well as high levels of adverse reactions, partially limited the therapeutic efficacy of PI3K/AKT/mTOR inhibitors. To increase therapeutic benefit, drug combinations and schedules need to be explored to identify those with the highest efficacy and lowest toxicity rate. In addition, defining appropriate patient subpopulations, for either monotherapy or drug combinations, and identifying predictive biomarkers remain a challenge.

**Abstract:**

Breast cancer is the most frequently diagnosed cancer and the primary cause of cancer death in women worldwide. Although early diagnosis and cancer growth inhibition has significantly improved breast cancer survival rate over the years, there is a current need to develop more effective systemic treatments to prevent metastasis. One of the most commonly altered pathways driving breast cancer cell growth, survival, and motility is the PI3K/AKT/mTOR signaling cascade. In the past 30 years, a great surge of inhibitors targeting these key players has been developed at a rapid pace, leading to effective preclinical studies for cancer therapeutics. However, the central role of PI3K/AKT/mTOR signaling varies among diverse biological processes, suggesting the need for more specific and sophisticated strategies for their use in cancer therapy. In this review, we provide a perspective on the role of the PI3K signaling pathway and the most recently developed PI3K-targeting breast cancer therapies.

## 1. Introduction

**Figure 1:**
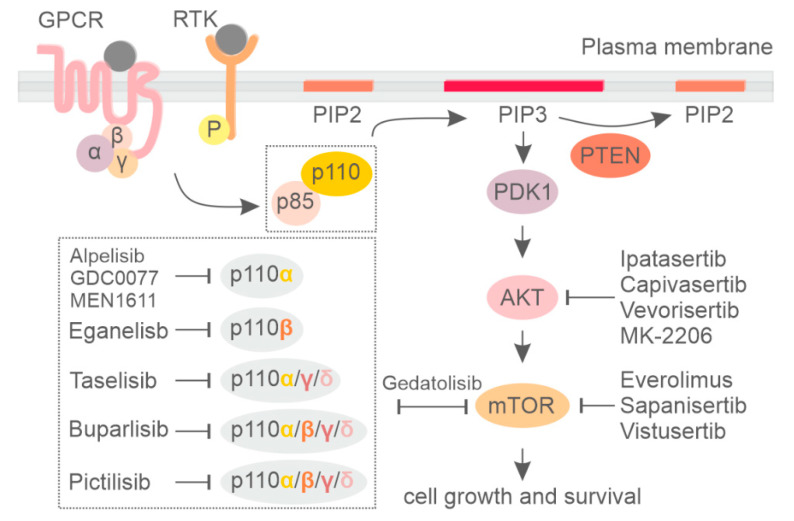
Signaling by the phosphatidylinositol-3-kinase (PI3K)/AKT/mammalian target of the rapamycin (mTOR) pathway and the respective inhibitors.

Phosphoinositide 3-kinase (PI3K) is a group of lipid kinases that phosphorylate the 3′-OH group of phosphatidylinositol (PI) at plasma and intracellular membranes. They are split into three different classes according to their structure, binding partners, and substrate specificity [1,2,3]. Among them, the most well-studied PI3K is class I PI3K, which generate PI(3,4,5)P3 (PIP3) starting from PI(4,5)P2 (PIP2). PIP3 is mainly produced at the plasma membrane in response to different stimuli and allows for the recruitment of a myriad of phospholipid effectors, including serine/threonine kinase AKT and 3-phosphoinositide-dependent protein kinase-1 (PDK-1), which are the central mediators of the PI3K pathway. PIP3 also facilitates PDK1 and AKT interaction, resulting in phosphorylation of AKT at thrPhosphorylated AKT promotes protein synthesis, cell growth, and cell survival and motility by activating many downstream kinases, including the mammalian target of the rapamycin (mTOR) complex [1]. On the other hand, the tumor suppressor phosphatase and tensin homolog (PTEN) dephosphorylates PIP3, counteracting PI3K signaling [4] (Figure 1).

Class I PI3K includes four highly homologous catalytic subunits, p110α, p110β, p110γ, and p110δ, which can associate with five regulatory subunits, collectively referred to as p85-type regulatory subunits (Figure 1) [5,6]. Whereas p110α and p110β isoforms are ubiquitously expressed, p110δ and p110γ expression is largely restricted to hematopoietic cells [7,8]. Dysregulation of phosphoinositide kinases, primarily in class IA PI3K, have been discovered in a number of human diseases, with mutations leading to either increased or decreased enzymatic activity being critically involved in cancer [9], developmental disorders [10], and primary immune deficiencies [11,12,13]. For an extensive description of the PI3K pathway, the reader can refer to the following reviews [1,14,15].

Several studies suggest that the PI3K/AKT/mTOR pathway is often genetically altered in human cancers [15,16]. Although many small molecule inhibitors targeting the PI3K/AKT/mTOR signaling pathway were pre-clinically studied, only some of the PI3K and mTOR inhibitors are currently approved for the treatment of human cancers in the clinic. Here we summarize the most recent advances in the inhibition of the PI3K/AKT/mTOR signaling pathway in breast cancer.

## 2. Genetic Alterations of the PI3K Pathway in Breast Cancer and Clinical Implications

The PI3K/AKT/mTOR pathway is frequently deregulated in breast cancer by different mechanisms, leading to increased PI3K activity and/or loss of PI3K inhibitory functions as well as mutation in tumor suppressor genes like INPP4B and PTEN phosphatases. Among PI3K genes, PIK3CA is one of the most frequently altered, with mutations occurring at two hotspot regions: an acidic cluster (E542, E545, and Q546) in the helical domain and a histidine residue (H1047) in the kinase domain. Whereas mutations on the helical domain mainly rely on the loss of p85-dependet inhibitory activity, activating mutations in the catalytic subunit of p110α directly stimulate lipid kinase activity by facilitating allosteric motions required for catalysis on membranes [17].

Mutations in the catalytic domain are the most frequent genetic alterations found in more than one third of early breast cancer tumors. In particular, PIK3CA activating mutations comprise up to 47% of HR+/HER2– (luminal A), 33% of HR+/HER2+ (luminal B), 39% of HR-/HER2+ (HER2-enriched), and 8–25% of basal-like/triple negative breast cancer subtypes [18,19,20,21,22,23,24,25]. The most common genetic alterations of PIK3CA were observed also in metastatic breast cancer biopsies, confirming the clonal character of these mutations [19,25,26]. Amplification of the PIK3CA gene locus has been also described [27], together with rare cases of PIK3CB amplification or PIK3R1 inactivating mutations [28,29].

The PI3KCB subunit also plays a key role in stimulating cell proliferation, invasiveness, and tumorigenesis in breast cancer [30,31]. A possible mechanism of PI3KCB activation in cancer occurs through the G protein-coupled receptor (GPCRs) [32]. In particular, a PI3KCB helical domain mutation (E633K) was first reported in an HER2-positive breast cancer patient [33]. E633K can enhance PI3KCB basal association with membranes, thus increasing PI3KCB activation [30]. PI3KCB has been shown to be responsible for the accumulation of PIP3 and reactivation of AKT in HER2-amplified breast cancers treated with a PI3KCA-specific inhibitor, and concomitant inhibition of PI3KCA and PI3KCB induces greater antitumor efficacy in HER2-amplified and PIK3CA mutant breast cancers [34].

Besides mutations in PIK3CA and PIK3CB genes, inactivating events also occur in tumor suppressors such as PTEN (Cancer Genome Atlas, 2012). Although the observed mutation rate for Luminal and HER+ is comparable to PIK3CA (up to 44% and 22%, respectively), the frequency of genetic alterations in PTEN observed in triple-negative subtypes reaches more than 65% [18,19,35,36,37]. Moreover, PTEN mutations select for an aggressive genomic subtype of ER+ breast cancer, with associated poor prognosis and acquired resistance to standard-of-care therapies [38]. The most studied cancer-associated PTEN mutations in its catalytic region are C124S and G129E, which abrogate PTEN phosphatase function. Knock-in mice models carrying either the C124S or G129E mutation were highly tumor prone and developed tumors in multiple tissues, including the thyroid, adrenal gland, gallbladder, prostate, and mammary glands, similar to what was observed in Pten+/− mice [38]. The fact that PIK3CA mutations and PTEN loss are nearly mutually exclusive implies that deregulated PIP3 is critical for tumorigenesis in breast cancers and that loss of PIP3 homeostasis by abrogation of either PIK3CA or PTEN relieves selective pressure for targeting of the other gene [39]. In addition to PTEN, inositol polyphosphate-4-phosphatase type IIB (INPP4B) can counteract PI3KCA signaling, and loss of its genetic locus has been reported in breast cancer [40,41]. Inactivation of the lipid phosphatase INPP4B is frequently observed in triple-negative breast cancer, where it functions as a tumor suppressor by regulating RTK trafficking and degradation. As a consequence, loss of INPP4B prolongs both PI3K and ERK activation [42]. In addition, recent findings suggest that INPP4B facilitates PI3KCA crosstalk with Wnt signaling in ER+ breast cancer via PI(3,4)P2-to-PI(3)P conversion on late endosomes, suggesting that these tumors may be targeted with combined PI3K and Wnt/β-catenin therapies [43].

Similarly, activating mutations in AKT1 occur in nearly 4% of Luminal [19,44], and genetic amplifications of AKT2 and PDK1 are observed in all breast cancer subtypes with a frequency of 3% and 20–38%, respectively [45,46]. Conversely, activating mutations or amplification of p70S6K or KRAS are infrequent events of unknown relevance in breast cancer, compared to other types of tumors [47,48].

Clinically, the implication of these molecular aberrations in breast cancer is still unclear and analysis of the prognostic role of PIK3CA mutation resulted in conflicting results [39,49,50,51,52,53,54,55,56,57,58,59]. Better invasive disease-free survival (IDFS [60]), but not distant disease-free survival (DDFS [60]) or overall survival (OS), was significantly associated with PI3KCA mutations in advanced-age, HR+, low-grade breast tumors [61]. PIK3CA mutations in operable primary breast cancer also indicated a significant correlation with better disease-free survival (DFS) [62] as well as a better recurrence-free survival in the Luminal A subtype [63].

However, PI3KCA mutations seem to have different clinical implications in advanced and metastatic breast cancer, resulting in chemotherapy resistance and poor outcome [64]. Progression-free survival (PFS) among cohorts of metastatic breast cancer using non-PI3K inhibitor-based therapies was reduced in PIK3CA-mutated compared with non-mutated patients [65,66,67,68,69,70,71]. In HER2+ breast cancer, mutations in PIK3CA are linked with poor prognosis not only in the advanced but also in the early setting [55,72]. On the other hand, the use of PI3K inhibitors in cohorts of breast cancer patients resulted in favorable PFS specifically for patients with PI3KCA mutation, thus showing that genetic alterations in PIK3CA are predictive markers of PI3K inhibitor benefit.

Moreover, in preclinical models, the hyperactivation of the PI3K/AKT/mTOR pathway is one of the major causes of the acquired resistance to endocrine therapy through ligand-independent activation of ER by its phosphorylation, mediated by the mTOR complex 1 (mTORC1)/S6K1 axis [73,74]. In HR+ breast cancer, acquired resistance to endocrine therapy can be abrogated by combination therapies targeting both ER and PI3K pathways. Although directly targeting PI3K and mTOR maximally inhibited hormone-independent cell growth and induced apoptosis, inhibition of signaling kinases upstream (IGF-IR/InsR/ErbBs) and downstream (mTOR) of PI3K also had partial inhibitory effects [73].

In the following paragraphs we will describe the clinical development and efficacy of different PI3K inhibitors for breast cancer treatment.

## 3. Clinical Usage of Pan-PI3K Inhibitors

- **Buparlisib**

Buparlisib (BKM120, Novartis Pharmaceuticals, Basel, Switzerland) is an orally available pan-PI3K inhibitor (Table S1, see supplementary file). In vivo studies showed a marked anti-proliferative and pro-apoptotic activity towards human breast cancer cell lines with PI3K pathway alterations, sufficient to induce a dose-dependent tumor growth delay or regression in PIK3CA-mutant xenografts [75]. Buparlisib safety and efficacy was then assessed in two large phase III randomized clinical trials called BELLE-2 and BELLE-During the BELLE-2 clinical trial, either buparlisib or placebo was administered in combination with fulvestrant to treat post-menopausal women with HR+/HER- metastatic breast cancer who had been previously treated or who had progressed on treatment with an aromatase inhibitor (AI) and up to one previous line of chemotherapy for advanced disease [67]. Similarly, BELLE-3 was designed to include Luminal A (HR+/HER-) breast cancer patients that had been previously unsuccessfully treated by prior endocrine therapy and mTOR inhibitors [68]. Both trials met their primary objective, demonstrating that the addition of buparlisib to fulvestrant increased PFS compared to fulvestrant alone. However, buparlisib was associated with significantly more grade 3/4 adverse events, including transaminitis, hyperglycemia, rash, and mood disturbance. Due to poor tolerability, many patients discontinued buparlisib prematurely, thus limiting the duration of the treatment. Particularly in BELLE-2, treatment discontinuation resulted in very short drug exposure (median 1.9 months), potentially limiting the efficacy of the combined therapy.

Buparlisib was also tested in combination with chemotherapy in breast cancer patients in the phase II/III clinical trial BELLE-4 by combining either buparlisib or placebo with paclitaxel as first-line treatment in HER- metastatic breast cancer patients [76]. Although effects of buparlisib appeared to be synergistic with paclitaxel in preclinical and clinical models [77], during this clinical trial, the addition of buparlisib to paclitaxel did not improve PFS in the full or PI3K pathway-activated study population. Consequently, the trial was stopped due to futility at the end of phase II. Also in this trial, buparlisib treatment resulted in a higher frequency of serious adverse effects, leading to a higher incidence of treatment discontinuation.

The toxicity profile of buparlisib observed in all the three clinical trials, including hepatic aminotransferase elevations and psychiatric complications such as depression, anxiety, and suicide attempts, strongly limited the potential for this drug to be adopted as standard of care. Nonetheless, the observation of significantly increased PFS in patients from BELLE-2 and BELLE-3 trials who had PIK3CA genetic alterations supports the rationale for PI3K inhibition plus endocrine therapy in HR+/HER2– Luminal A breast cancer patients.

- **Pictilisib**

Pictilisib (GDC-0941; Genentech, San Francisco, CA, USA) is a pan-PI3K inhibitor showing in vitro equipotent inhibitory effects on p110α and p110δ isoforms and fewer inhibitory effects on p110β and p110γ isoforms [78] (Table S1). Its efficacy was evaluated in two phase II clinical trials called FERGI and PEGGY [69,79]. In the FERGI trial, either pictilisib or placebo was administered in combination with fulvestrant in postmenopausal women with ER+/HER2− metastatic breast cancer resistant to treatment with aromatase inhibitor [69]. During the first part of the trial, patients were recruited independently from their PIK3CA mutational status, whereas during the second part, patients without PIK3CA mutations were excluded. Similar to the buparlisib treatment, high-grade adverse effects occurred in more than half of the enrolled patients. Moreover, no significant difference in media PFS between pictilisib and the placebo was observed.

The second trial, PEGGY, was designed to include randomized pre- and post-menopausal Luminal A patients (HR+/HER2–) to receive paclitaxel with either pictilisib or placebo [79]. In both groups, one third of recruited patients was characterized by genetic alterations in the PIK3CA gene. However, no significant differences in terms of PFS or over all response were observed between the two groups, nor concerning PIK3CA mutational status.

In light of the results from these clinical trials, which were far from achieving clinical benefit, the authors suggested that future developments need to investigate inhibitors with marked selectivity towards PI3K-specific isoforms or mutants, thus improving tolerability and providing the strongest and safest therapeutic index.

## 4. PI3K Isoform-Specific Inhibitors

Considering the limitations encountered with pan-PI3K inhibitors, the selective inhibition of specific PI3K isoforms permits the administration of therapeutic doses of drugs, avoiding severe off-target toxicity. On the other hand, a selective approach demands a precise strategy to select patients who may benefit from that treatment [80]. In breast cancer, activating mutations in the PIK3CA gene are the most frequent alteration of the PI3K pathway, leading to hyperactivation of p110α. Therefore, great efforts have been directed to developing PIK3CA-selective inhibitors to specifically target this PI3K isoform.

- **Alpelisib**

Alpelisib (BYL719) is the first oral PI3K inhibitor selectively targeting the p110α isoform (Table S1). Its efficacy was first assessed in preclinical models showing potent inhibition over the two most common PIK3CA mutations (H1047R and E545K) at nanomolar concentration (4.6 nM/L) [81]. Notably, treatment with alpelisib not only interfered with PIK3CA-mediated downstream signaling, but also induced a dose-dependent decrease in p110α protein levels in ER+/PIK3CA-mutated breast cancer cell lines [82], suggesting a dual mode of action. The combination of alpelisib with fulvestrant also demonstrated synergism between the two drugs in xenograft models [73]. A tolerable safety profile and encouraging activity in patients with PIK3CA-altered solid tumors was reported for alpelisib in a first-in-human phase I study [28]. The subsequent phase II clinical trial was conducted to assess the maximum tolerable dose, safety, and efficacy of alpelisib in combination with fulvestrant in HR+/HER2– metastatic breast cancer [83]. Partial or complete response was observed among 29% of pretreated metastatic breast cancer patients with PIK3CA alterations, whereas no tumor response was reported in the PIK3CA wild-type group [83]. A favorable safety profile in these patients included mainly on-target effects such as hyperglycemia, nausea, and diarrhea [83].

Next, a phase III SOLAR-1 clinical trial evaluated the efficacy and safety of alpelisib in combination with hormonal therapy (fulvestrant) in HR+/HER2– metastatic breast cancer patients who recurred or progressed after endocrine therapy [65]. Patients were selected and stratified based on the PIK3CA mutational status to also include a cohort of PIK3CA wild type as proof of activity in this subgroup. PIK3CA status was determined by tumor tissue RT-PCR and led to the inclusion of 341 patients in the PIK3CA-mutant cohort and 231 in the wild-type group. The primary and secondary endpoints for this study were to evaluate the PFS and the overall survival in patients with PIK3CA genetic alterations together with the safety and efficacy in the PIK3CA wild-type group. The median PFS of patients with PIK3CA genetic alterations was 11.0 months in the alpelisib/fulvestrant arm versus 5.7 months in the placebo/fulvestrant arm (HR 0.65 95% CI 0.50–0.85; *p* < 0.001). The overall response was also higher with alpelisib/fulvestrant compared to placebo/fulvestrant (26.6% and 12.8%, respectively). Conversely, in the PIK3CA wild-type group, alpelisib administration was not significantly associated with improved PFS (7.4 versus 5.6 months, respectively; HR 0.85; 95% CI, 0.58–1.25). Toxicity due to alpelisib administration was associated with specific p110α inhibition and included hyperglycemia (63.7% versus 9.8% for the alpelisib and placebo arms, respectively), diarrhea (57.7% versus 15.7%, respectively), and rash (35.6% versus 5.9%, respectively). Permanent discontinuation due to AEs occurred in 25% of patients in the alpelisib group versus 4.2% in the placebo arm. The positive results from the SOLAR-1 trial prompted the Food and Drug Administration (FDA) to approve the combination of alpelisib with fulvestrant for the treatment of men and postmenopausal women with HR+/HER2–, PIK3CA-mutated, advanced, or metastatic breast cancer, as detected by an FDA-approved test following progression on or after an endocrine-based regimen. One year after the approval of alpelsib, the data of overall survival were released. Although overall survival with a median follow-up of 30.8 months did not meet statistical significance, the absolute difference of 8 months observed between the alpelisib versus the placebo treatment was clinically relevant and valuable, with PFS benefit not only maintained but also increased in terms of overall survival outcome [84].

The SOLAR-1 clinical trial started to recruit the first patients during the second half of 2015, a few months after the FDA granted accelerated approval for the CDK4/6 inhibitor palbociclib in combination with endocrine treatment for postmenopausal HR+/HER2- metastatic breast cancer [85,86]. For this reason, only 5% of patients with mutations in PIK3CA included in SOLAR-1 had received a CDK4/6 inhibitor before being enrolled in the clinical trial. To better evaluate the efficacy of alpelisib in patients treated with a CDK4/6 inhibitor, the phase II BYLieve trial was designed [87]. In this trial, patients were enrolled based on their previous treatment with an aromatase inhibitor in combination with CDK4/6 and received fulvestrant plus alpelisib. Almost 50% of patients showed no disease progression at 6 months. Median PFS also resulted in 7.3 months [87], in line with previous results from the SOLAR-1 subgroup analysis in which 44% of patients (9 out of 20) receiving fulvestrant plus alpelisib were alive without disease progression at 6 months and a median PFS of 5.5 months [65]. These findings support the use of alpelisib in combination with fulvestrant after CDK4/6 inhibitors [65,87].

- **Taselisib**

Taselisib (GDC-0032, Genentech, San Francisco, CA) is an oral PI3K inhibitor equally inhibiting p110α, δ, and γ isoforms of class I PI3K but with 30-fold less potency against p110β [72] (Table S1). Given its greater selectivity against PI3K isoforms, taselisib was expected to have improved efficacy on PIK3CA-mutant tumors and less toxic effects compared to pan-PI3K inhibitors. In particular, treatment with taselisib resulted in marked tumor suppression in preclinical studies performed on PIK3CA-mutant xenografts [88]. An initial phase I clinical trial demonstrated clinical activity of taselisib in patients with advanced solid tumors, particularly in breast cancers with PIK3CA genetic alterations, with an overall response of 36% compared to no response in patients with wild-type PIK3CA [89]. Based on encouraging phase I results, a phase III clinical trial called SANDPIPER was performed on postmenopausal ER+/PIK3CA-mutated metastatic breast cancer patients previously treated with AI. Fulvestrant was administered in combination with either taselisib or placebo and the primary endpoint was the assessment of PFS in patients with PIK3CA-mutated tumors (>80% of participants) [90]. Median PFS was significantly longer, although modest, in the taselisib arm (7.4 months) versus the placebo arm (5.4 months) (HR 0.7 95% CI 0.56–0.89; *p* = 0.004). Patients treated with taselisib also had a significantly higher objective response rate compared to placebo (28% and 11.9%, respectively). Treatment with taselisib was also associated with higher severe adverse effects, including diarrhea (grade 3/4 of 12% for taselisib arm versus <1% for placebo) and hyperglycemia ((grade 3/4 11% versus <1%, respectively). Because the clinical benefits observed in the SANDPIPER trial were modest and the tolerability was questionable, further investigation of taselisib was stopped [90]. One of the reasons accounting for the lack of efficacy of taselisib is likely related to the less potent and specific inhibition of p110a compared to alpelisib, as evidenced by the higher rates of hyperglycemia in the SOLAR-1 trial compared to the SANDPIPER trial [81,91].

## 5. PI3K Pathway Inhibition in HER2+ and Triple-Negative Breast Cancer Subtypes

- **HER2-Positive Breast Cancer**

Preclinical studies demonstrated that HER2 signaling largely relies on p110α rather than on other class-I PI3K isoforms [92], thus providing a strong rationale for therapeutic intervention and targeting of PIK3CA in HER2+ breast tumors. Particularly, reduced pathological complete response (pCR) rate was linked to PIK3CA mutational status in HER+ breast cancer patients who received neoadjuvant chemotherapy and anti-HER2 therapy [93]. Some clinical trials were conducted to determine the potential benefit of inhibiting PI3K in HER2+ breast tumors. A phase I study called PIKHER2 was designed to assess the effect of combining pan-PI3K inhibitors buparlisib and lapatinib in trastuzumab-resistant HER2+ metastatic breast cancer independently of PIK3CA mutational status [94]. The observed clinical benefit rate (CBR) was 29%, and complete response was observed in one patient (4%). Another phase Ib/II clinical trial tested the combination of buparlisib with trastuzumab in HER2+ breast tumors resistant to trastuzumab [95]. Also in this case, the trial was conducted without considering the PIK3CA mutational status. Although the authors evidenced some clinical activity with the combination (2% complete response and 8% partial response), the trial failed to reach the estimated primary endpoint of objective response rate >25%.

The NeoPHOEBE phase II clinical trial enrolled HER2+ early breast tumors to be treated with either buparlisib or placebo in combination with paclitaxel and trastuzumab [96]. In this setting, the percentage of patients with genetic alteration in PIK3CA was below 20%. The authors observed a pCR rate of 32% for the buparlisib group compared to 40% in the placebo group. In line with other trials conducted on buparlisib, its administration was associated with higher toxicity, leading to 36% of adverse events compared to less than 10% in the placebo arm.

Besides pan-PI3K inhibitors, a phase I trial was conducted with the p110α-specific alpelisib inhibitor in association with trastuzumab emtansine (TDM-1) in trastuzumab-resistant breast cancer patients [97]. The objective response rate of this study was 43%, with 60% of clinical benefit rate specifically in patients who had previously progressed on TDM-Moreover, 53% of patients included in the study presented alteration of the PI3K pathway, including PIK3CA mutations, PTEN loss, or AKT overexpression. Almost half of these patients showed clinical benefit rate, even in case of previous progression in TDM-1 therapy [97]. Adverse effects (grade > 3) occurred in 59% of patients but they were generally manageable. These findings demonstrated that activation of the downstream PI3K pathway can be a possible mechanism of tumor resistance to TDM-1 [98].

Other alpha-specific class I PI3K inhibitors are currently being tested in clinical trials to target the PI3K pathway in breast cancer patients. Among them, GDC-0077 is a new potent, orally available, and p110α-selective inhibitor. It has already shown robust activity in preclinical models of breast tumors with genetic alterations in PIK3CA [99,100]. Mechanistically, GDC-0077 leads to downregulation of p110α, thus interfering with the activation of PI3K downstream targets such as the phosphorylation of AKT. Accordingly, treatment of human PIK3CA-mutant breast cancer cell lines with GDC-0077 resulted in reduced proliferation and increased apoptosis. Similar results were observed in xenograft models where GDC-0077 was combined with standard-of-care treatments for HR-positive breast cancer such as anti-estrogen (fulvestrant) or CDK4/6 inhibitor (palbociclib) [100]. An ongoing phase I trial showed that GDC-0077 in association with palbociblib and fulvestrant can be combined at maximum doses. INAVO120 is a phase III, randomized, double-blind, pbo-controlled study that will assess the efficacy and safety of GDC-0077/pbo plus palbociblib and fulvestrant in patients with PIK3CA-mutant/HR+/HER2– advanced metastatic breast cancer [101].

Another novel PI3K inhibitor, targeting the mutated form of p110α and p110γ, is MEN1611 [102]. In both xenografts and PDX models of breast cancer, MEN1611 showed significant activity either as a monotherapy or in combination with targeted therapies in breast cancer and other solid tumors. In HER2+ breast cancer cell lines mutated for PIK3CA, as well as in patient-derived xenograft models, MEN1611 seemed to act synergistically when associated with trastuzumab, also inducing a dose-dependent p110α protein depletion and a pro-inflammatory phenotype compatible with p110γ inhibition [103].

- **Triple-Negative Breast Cancer (TNBC)**

In the other types of breast cancer, there is a shortage of clinical trials of alpelisib application. For example, in triple-negative breast cancer (TNBC), a phase I clinical trial is testing the effect of chemotherapy combining alpelisib with enzalutamide in AR+ and PTEN+ breast cancer, including a cohort of TNBC (NCT03207529). Another phase III study is assessing the efficacy and safety of alpelisib plus nab-paclitaxel in subjects with advanced TNBC with PIK3CA mutation. The results from these ongoing trials will provide us a better perspective on how alpelisib affects triple-negative breast cancer patients. Similar results have been reported in a phase II neoadjuvant-based clinical trial (NCT02273973).

A phase I clinical trial (NCT01884285) is also studying the PI3KCB/PI3KCD inhibitor AZD8186 in patients with TNBC and known PTEN-deficient/-mutated or PIK3CB-mutated/-amplified advanced tumors and in combination with abiraterone acetate or AZD2014, an mTOR inhibitor [104]. AZD8186 has single-agent efficacy in PTEN-deficient TNBC cell lines in vitro, but has limited single-agent efficacy in vivo [105]. However, AZD8186 showed enhanced efficacy when combined with paclitaxel and anti-PD1 in vivo [105]. Further study is needed to determine the optimal combination therapies for PTEN-deficient breast cancer.

Immuno-oncology is also gaining increasing interest as a valuable therapeutic strategy in breast cancer [15,106]. TNBC is considered the most immunogenic subtype of breast cancer, with a higher lymphocyte infiltration rate than HER2+ or HR+ tumors and thus is regarded as a promising target for immunotherapies [107]. MARIO-3 (NCT03961698) is a phase 2 clinical study designed to evaluate IPI-549 (eganelisib), Infinity Pharmaceutical’s oral immuno-oncology product targeting immuno-suppressive tumor-associated myeloid cells through selective inhibition of PI3KCG, in combination with Tecentriq (atezolizumab) and Abraxane (nab-paclitaxel) in front-line TNBC. The novel triplet regimen of IPI-549, atezolizumab, and nab-paclitaxel showed promising antitumor activity irrespective of biomarker status, with manageable toxicity. The expansion phase of the phase II study is currently enrolling, with a target completion date of 2022 [108].

## 6. Currently Available Inhibitors Acting on AKT and mTOR in Breast Cancer

- **AKT Inhibitors**

AKT consists of three isoforms (AKT1, AKT2, and AKT3). It is the major downstream target of PI3K and one of the most common molecular alterations in cancer [109]. Targeting of this altered pathway by pharmacologic modulation of AKT activity represents a powerful strategy for cancer intervention [110]. Among different AKT inhibitors (Table S1), AZD5363 (capivasertib) has been used as a monotherapy in breast cancer in a phase I, open-lab study for patients with AKT E17K mutations [111]. Capivasertib was well tolerated and achieved plasma levels and robust modulation of AKT activity in tumors. Proof-of-concept responses were observed in patients with PIK3CA-mutant cancers treated with AZD5363 [111]. Another pan-AKT inhibitor, GDC-0068 (ipatasertib) has been used as a monotherapy in triple-negative breast cancer cases and has already entered phase I and II studies [112]. Dose-limiting side effects during treatment together with dose reduction occurred in both trials and were mainly due to the fact that the ATP-binding pocket of AKT is highly conserved among other kinases, which limits selectivity [113].

Major efforts are now directed towards the identification of AKT-specific and isoform-selective small molecules. For instance, MK-2206 and miransertib (ARQ092) are bioactive allosteric inhibitors that offer greater specificity, reduced side effects, and lower toxicity compared to other targeted approaches [109,114]. In a clinical trial (I-SPY TRIAL, NCT01277757), MK-2206 is currently tested in combination with or without trastuzumab for treatment of advanced breast cancer with PIK3CA or AKT mutations, and/or PTEN loss/PTEN mutation [115]. However, MK-2206 monotherapy had limited clinical activity in advanced breast cancer patients due to tumor heterogeneity and tolerable dose. Similarly, MK-2206 is unlikely to add further benefit to the efficacy of anastrozole alone in a phase II study based on PIK3CA-mutant ER+ breast cancers (NCT01776008). Future study designs should consider emerging data regarding population subtypes that may benefit most from specific drug combinations.

Another phase 1b study of the miransertib next-generation inhibitor ARQ 751 (vevorisertib, NCT02761694) as a single agent or in combination with either paclitaxel or fulvestrant in patients with advanced solid tumors with PIK3CA/AKT/PTEN mutations was recently completed, although results are not yet available. The pan-inhibitor MK2206 remains the most prominent of the allosteric inhibitors; however, others such as TAS-117 have also shown promising effects [109,116,117].

Another innovative approach to targeting AKT in disease involves the irreversible covalent modification of two noncatalytic cysteines in the activation loop of AKT by covalent–allosteric inhibitors (CAAIs), such as borussertib. The in vivo efficacy of borussertib was proven in combination studies with MEK-inhibitor trametinib in KRAS-mutant patient-derived xenograft models, leading to a partial response [114,118]. Further studies are required to better understand its clinical relevance, particularly in breast cancer.

- **mTOR Inhibitors**

mTOR is one of the most important downstream effectors of the PI3K/AKT pathway. Inhibitors targeting mTOR, including everolimus (RAD001), MLN0128, and AZD014, have been broadly studied and evaluated in hematological cancer and solid tumors [119] (Table S1). Everolimus and its combination with exemestane has been approved by the FDA for the treatment of hormone receptor-positive/HER2-negative (HR+/HER2−) breast cancer [120,121]. This synergistic effect was also observed in postmenopausal women with metastatic ER+/HER− breast tumor. In this study, a combination of everolimus and tamoxifen showed a significant reduction in cancer progression and increased overall survival rate compared to tamoxifen monotherapy [122]. Similarly, a clinical study including ER+ breast cancer patients showed that treatment with neoadjuvant letrozole and everolimus before surgery resulted in higher clinical response and reduced tumor proliferation compared to letrozole alone [123].

Sapanisertib (MLN0128) is an oral, potent, and highly selective ATP-competitive inhibitor of mTOR kinase that exhibits dual specificity against both mTOR complexes (mTORC1 and mTORC2). In a phase II study (NCT02049957), sapanisertib plus exemestane or fulvestrant was well tolerated and exhibited clinical benefit in postmenopausal women with pretreated everolimus-sensitive or everolimus-resistant breast cancer [124,125]. A randomized study of AZD2014 (vistusertib) in combination with fulvestrant in metastatic or advanced breast cancer (MANTA, NCT02216786) was conducted. The combination of fulvestrant and everolimus demonstrated significantly longer PFS compared to fulvestrant and vistusertib or fulvestrant alone. The trial failed to demonstrate a benefit of adding the dual mTORC1 and mTORC2 inhibitor vistusertib to fulvestrant [126].

mMTOR inhibitors were generally well tolerated in clinical trials. The most frequently observed side effects included headache, fatigue, and erythema (skin rash). In particular, the use of MTOR inhibitors was associated with a higher risk of developing hypertriglyceridemia, hypercholesterolemia, and hyperglycemia [116,127,128,129]. Future studies should take into account the improvement of clinical benefits together with reduced risk of adverse events.

- **Dual PI3K/mTOR Inhibitors**

During the early developmental phases of mTOR and PI3K inhibitors, it was noted that the catalytic pocket of these two enzymes possess structural similarities, making it possible to design ATP-competitive drugs targeting both kinases simultaneously [130,131]. In particular, mTOR inhibition commonly results in the repression of a negative feedback loop, which activates the PI3K and MAPK pathways. In line with this, inhibition of both PI3K and mTOR was proposed as a good strategy to limit this compensatory mechanism [132,133]. In breast cancer, gedatolisib (PF-05212384) is a dual PI3K/mTOR inhibitor that was evaluated in combination with either docetaxel, cisplatin, or dacomitinib in triple-negative breast cancer (NCT01920061). This phase I study assessed the safety, pharmacokinetics, and pharmacodynamics of these combinations in patients with advanced cancer in order to determine the maximum tolerated dose in each combination. The cisplatin combination expansion portion was used to evaluate the anti-tumor activity of gedatolisib plus cisplatin in patients with TNBC in two separate arms. A manageable toxicity profile was observed in gedatolisib combined with docetaxel, cisplatin, or dacomitinib. Dose escalation to determine the maximum tolerated dose is still ongoing [134]. Another phase I study was conducted to assess the tolerability and clinical activity of gedatolisib in combination with either palbociclib/letrozole or palbociclib/fulvestrant in women with metastatic breast cancer (NCT02684032). This clinical trial was recently concluded; however, results are not yet available. Gedatolisib combined with either palbociclib/letrozole or palbociclib/fulvestrant showed manageable toxicity and promising antitumor activity. Further analysis on dose escalation is being completed and dose expansion is ongoing [135]. Whether the effect of this class of agents in combination with immunotherapy can lead to further clinical benefit is an open issue.

## 7. Rationale for Targeting Class II PI3K in Breast Cancer

Class II PI3Ks consist of three genes encoding for distinct functional isoforms: PI3K-C2α and PI3K-C2β, which are ubiquitously expressed [2,136], and PI3K-C2γ, whose expression is mainly restricted to liver [137,138]. Different from class I, class II PI3Ks act as monomers, regulating vesicle trafficking and membrane remodeling through their conserved N-terminal domain [2,139,140]. They synthetize PI(3)P on endosomes and PI(3,4)P2 at plasma membrane [138,139,141,142,143,144,145,146,147]. However, their catalytic pocket is structurally different from class I PI3K and largely unaffected by treatments with class I PI3K inhibitors [148,149,150]. Recent studies suggested that class II PI3Ks are directly involved in breast cancer progression independently of class I PI3K, opening the way for the development of new therapeutic strategies targeting this enigmatic class of PI3K in breast cancer.

PI3K-C2α is the most studied isoform, and it has been linked with breast cancer in different studies. Overexpression of the PI3K-C2α encoding gene, PIK3C2A, was found in an MCF7 cancer stem-cell side population, correlating with increased tumorigenesis in mouse models [151]. This suggests that PI3K-C2α might have a role in the early phases of cancer development. Conversely, PI3K-C2α was found to rarely be mutated in breast cancer patients on publicly available datasets, but it was observed as lost at both the mRNA and protein levels in a large cohort of breast cancer patients [152]. This study demonstrated that PI3K-C2α has kinase-independent activity by stabilizing microtubules at kinetochore during mitotic metaphase and allowing proper chromosome congression [152]. Loss of this activity was associated with increased genomic instability that led to the emergence of fast-growing clones with mitotic checkpoint defects. Therefore, low PIK3C2A expression was related to high sensitivity to paclitaxel treatment in human breast cancer patients [152]. Accordingly, development of future inhibitors targeting PI3K-C2α scaffold function in breast cancer can be beneficial in combination with microtubule-targeting drugs, i.e., paclitaxel.

Different from PI3K-C2α, PI3K-C2β isoform was found to be overexpressed in human primary breast tumors and in lymph-node metastases compared to non-neoplastic breast tissue [153]. An additional study conducted on different human breast cancer cell lines found a similar increase in PI3K-C2β levels, which was directly related to enhanced tumorigenesis and invasive abilities both in vitro and in vivo [153]. In line with this, PI(3)P produced by PI3K-C2β was shown to be involved in breast cancer migration and invasion by dismantling lamellipodia and filipodia, thus resulting in reduced cell adhesion [154,155,156]. Consistently, data from xenograft models showed that the overexpression of PI3K-C2β leads to increased cell motility and enhanced metastasis development in vivo [153].

At the current state, there is no pharmacological option to selectively target the class II PI3K that has been clinically tested. Nevertheless, given their emerging functions in many pathological processes, some efforts have been made to find effective inhibitors targeting this less investigated class of PI3K. In general, pharmacological inhibition of class II can be achieved by “off-target” activities of different class I inhibitors [157], such as PIK90, PIK124, PI-103 [158], and NVP-BEZ235 [159]. Of note, some inhibitors, such as PI701 and PI702 [157,160], have shown to be more selective for PI3K-C2β than PI3K-C2α isoform. However, a lack of selectivity for class II keeps these options far from being effective at specifically targeting class II PI3K in any pathological process. Interestingly, a subsequent study claimed the discovery of a PIK3-C2α-selective inhibitory molecule, MIPS-21335, suggesting a potential new therapeutic anti-thrombotic approach based on class II PI3K-selective targeting [161].

Taken together, recent discoveries showed that PI3K activity in cancer development and migration is not limited to PIP3 production by class I PI3K, thus highlighting the importance of class II PI3K-derived phosphoinositides. These findings suggest that pharmacological targeting of class II PI3K may lead to the development of alternative therapeutic strategies for treating breast cancer, emphasizing the need for class II PI3K-selective inhibitors in clinic.

## 8. Conclusions

Great effort has been directed to demonstrating the relevance of targeting the PI3K pathway in breast tumors driven by PIK3CA aberrations. Although pan-PI3K inhibitors showed efficacy in PIK3CA-mutated patients, particularly in combination with endocrine therapy, their low tolerability due to lack of isoform selectivity largely limits their clinical usage. The development of PI3K isoform-specific inhibitors such as alpelisib was able to partially overcome these issues, providing new treatment opportunities for HR+/HER2–, PIK3CA-mutated, metastatic breast cancer that progresses after endocrine therapy. However, proper adverse-event management is also required for alpelisib to limit patients’ discontinuation and dose reduction, which were events frequently observed during trials. Further clinical studies to evaluate combinations of hormone therapy with PI3K, AKT, mTOR, or CDK 4/6 inhibitors, together with clinical trials in other breast subtypes, are still ongoing and will lead to improved therapies to treat breast cancer patients. In addition, further studies may lead to the emergence of a new class of PI3K inhibitors selectively targeting class II PI3K.

## Supplementary Materials

The following are available online at https://www.mdpi.com/article/10.3390/cancers13143517/s1, Table S1: selective inhibitors of PI3K/AKT/mTOR pathway in breast cancer.
